# Correlation of Hemoglobin, Albumin, Lymphocyte, and Platelets (HALP) Score With Outcome of Acute Ischemic Stroke

**DOI:** 10.7759/cureus.67979

**Published:** 2024-08-27

**Authors:** Nithesh Babu Ramesh, Abinaya Srinivasa Rangan, Gnanadeepan Thirugnanam, Mahendra Kumar Kalappan

**Affiliations:** 1 General Medicine, Saveetha Medical College and Hospitals, Saveetha Institute of Medical and Technical Sciences, Saveetha University, Chennai, IND

**Keywords:** mortality, immunohematological markers, clinical predictors, ischemic stroke prognosis, stroke outcome, stroke risk factors, acute ischemic stroke

## Abstract

Background: Acute ischemic stroke (AIS) stands as a primary cause of both mortality and enduring disability. Given its significant impact on public health, efforts are ongoing to improve prognostic instruments and treatment approaches for this illness. In recent years, attention has been drawn towards the exploration of novel indicators of hemoglobin, albumin, lymphocyte, and platelets (HALP) that measure systemic inflammation and nutritional status. This study aims to investigate the relationship between the HALP score and the outcome of an acute ischemic stroke.

Methodology: This prospective observational study was conducted at a tertiary care hospital, with approval from the Institutional Ethical Committee. A total of 100 AIS patients were enrolled in the study, and their demographic details were collected. Within 24 hours of stroke onset, laboratory tests were performed, including measurements of albumin, hemoglobin, lymphocytes, and platelet count. The HALP scores of these patients were recorded. The severity of AIS was assessed using the National Institutes of Health Stroke Scale (NIHSS) upon admission.

Results: This study comprised 100 patients, with 66% and 44% being male and female, respectively, with a standard deviation of age 63. Following 28 days of in-hospital care and a 90-day outpatient follow-up, 16 patients were categorized as the non-survival group. The results showed a significant difference in hemoglobin values in the admitted groups (p = 0.04). Lymphocyte counts were higher in survivors compared to non-survivors with non-significant (p = 0.105). Furthermore, the HALP score of survivors is (a median of 34.0) and for non-survivors is (a median of 24.0), which showed a significant difference with a p-value of 0.022 and is statistically significant.

Conclusion: Lower HALP score is associated with increased mortality in stroke patients. The HALP score emerges as a potentially valuable predictor of mortality in critically ill patients. This underscores the importance of considering the HALP score in prognostic assessments and treatment decisions for individuals affected by stroke.

## Introduction

Stroke is characterized by a neurological deficit arising from an acute focal injury to the central nervous system due to vascular factors [1]. It stands as the third most prevalent cause of mortality and a prominent contributor to disability in both developed and developing nations, profoundly affecting the quality of life for patients and their families while exacerbating the societal burden [2]. According to the World Stroke Organization, there are over 13.7 million reported stroke incidents each year, leading to more than 2.7 million deaths from stroke attacks. Acute ischemic stroke (AIS) is more common, making up to 85% of cases [3]. In AIS, inflammation is recognized as a significant component in both development and progression. Vascular occlusion leads to the formation of necrotic cells, subsequently triggering an inflammatory response.

Furthermore, an unfavorable prognosis in AIS is linked to factors such as irregular blood clotting, inadequate nutritional status, and the existence of inflammation [4]. By exploring novel indicators, there is a potential to enhance the early identification of high-risk patients with unfavorable outcomes [5]. Since its introduction, the hemoglobin, albumin, lymphocyte, and platelet (HALP) score has gained attention as an innovative prognostic biomarker capable of predicting various clinical outcomes across numerous types of cancers [6]. Recent studies indicate its potential to predict stroke recurrence and poor outcomes [7]. Anemia is prevalent and an independent predictor of mortality in AIS cases. Serum albumin plays a neuroprotective role, and hypoalbuminemia is linked to poor outcomes. Lymphocytes play a crucial regulatory role in post-acute ischemic stroke inflammation and are indicative of exacerbating ischemic brain injury and declining neurological function [8]. Platelet hyperactivity increases the risk of developing atherosclerotic vascular complications [9]. In a study conducted by Xu et al., a lower HALP score was related to a higher risk of early-onset post-stroke cognitive impairment [10]. The HALP score is a readily calculable indicator of systemic inflammation and healthy status [11]. However, limited studies have shown the association between the HALP score and AIS. Therefore, this study aimed to evaluate the relationship between the HALP score and the outcomes of AIS.

## Materials and methods

This prospective cross-sectional study was meticulously conducted at the Department of General Medicine, Saveetha Medical College and Hospital, India over twelve months from March 2023 to March 2024. The primary objective was to investigate patients who had suffered from acute ischemic stroke (AIS). By focusing on this specific patient population, the study aimed to elucidate the relationship between various hematological parameters and the severity of AIS. The structured and time-bound nature of the study ensured a comprehensive and methodical approach to data collection and analysis.

The study cohort comprised 100 patients, all of whom were admitted to the department with a confirmed diagnosis of AIS during the study period. Stringent inclusion and exclusion criteria were employed to ensure the reliability and relevance of the findings. Only patients aged between 18 and 80 years with a confirmed diagnosis of AIS were included. Exclusion criteria were meticulously defined to eliminate confounding variables and included patients with hemorrhagic stroke, those transferred from other hospitals, individuals with missing laboratory data, and patients suffering from severe infectious diseases or malignancies.

Ethical approval was a cornerstone of this study, obtained from the Institutional Ethical Committee prior to commencement. This approval underscored the study's commitment to adhering to ethical guidelines and ensuring patient safety. The study was designed to pose minimal risk to the participants, with all procedures and interventions conducted in compliance with established medical and ethical standards. This ethical rigor ensured the integrity of the research process and the validity of the findings.

Comprehensive demographic and clinical data were collected for each patient. Demographic information, including age and gender, was recorded. Baseline clinical data encompassed a detailed medical history, blood pressure readings, pulse rate, and information on any medications the patient was taking prior to the stroke. The severity of AIS was assessed upon admission using the National Institutes of Health Stroke Scale (NIHSS) [12], a widely recognized and validated tool for evaluating stroke severity. This holistic data collection approach provided a robust dataset for subsequent analysis.

Within the first 24 hours of stroke onset, fasting blood samples were obtained from each patient for laboratory analysis. The use of fasting samples ensured the accuracy and consistency of the biochemical measurements. An automated biochemical analyzer (SYSMEX XN 1000) was employed to determine serum levels of key hematological parameters: albumin, hemoglobin, lymphocytes, and platelets. These parameters were chosen for their potential relevance to stroke outcomes. The HALP score, a composite index of these parameters, was calculated using the formula: (hemoglobin (g/L) × albumin (g/L) × lymphocytes (L))/platelets (10^9^/L). This score was hypothesized to correlate with AIS severity and patient prognosis.

The collected data were subjected to rigorous statistical analysis. Quantitative variables, such as age and the various hematological parameters, were summarized using median values and interquartile ranges (IQRs), providing a clear depiction of the data distribution. Categorical variables, including gender and the presence of specific medical conditions, were presented as frequencies and percentages. The Mann-Whitney and Chi-square tests were the statistical tests that were used in this study. Statistical significance was assessed using a significance level of 0.05. This comprehensive statistical approach enabled the identification of meaningful patterns and relationships within the data, contributing to a nuanced understanding of the factors influencing AIS severity and outcomes. The use of advanced statistical methods ensured the robustness and reliability of the study’s conclusions.

## Results

According to the demographic data, the median age of the total population was 63.0 years, with no significant variance between survivors and non-survivors (p = 0.586). A higher proportion of females was observed among non-survivors (10 out of 16) compared to survivors (36 out of 84). Significant differences were noted in systolic blood pressure (SBP), with non-survivors exhibiting a lower SBP compared to survivors (p = 0.045). However, no significant disparity was found in diastolic blood pressure (DBP) between the groups (p = 0.324). While pulse rates tended to be higher in non-survivors than survivors, this contrast did not achieve significance (p = 0.15), as shown in Table 1.

**Table 1:** The demographic profiles of patients categorized by survivors and non-survivors. The statistical tests used here are the Mann-Whitney test and the Chi-square test. IQR: interquartile range.

Variables	Total (n = 100)	Survivors (n = 84)	Non-survivors (n = 16)	P-value
Age in years (median-IQR)	63.0 (55.20–77.5)	64.35 (55.20–78.0)	63.0 (55.20-76.26)	0.586
Gender				
Female (proportion)	44	36	10	0.132
Male (proportion)	56	48	6	
Systolic BP in mm Hg (median-IQR)	139 (132-154)	140 (132-154)	126 (114-140)	0.045
Diastolic BP in mm Hg (median-IQR)	83 (72-91)	83 (75-91)	75 (72-92)	0.324
Pulse rate (median-IQR)	78 (70-92)	78 (70-90)	85 (75-98)	0.15

In the current investigation, non-survivors exhibited a significantly higher median WBC count (12.31 × 10^3^/μL) compared to survivors (9.32 × 10^3^/μL), suggesting a potential association between elevated WBC and increased mortality (p = 0.001). A significant difference was observed in hemoglobin levels between survivors and non-survivors (p = 0.046), with both groups having similar median values (128-130 g/L). Lymphocyte counts were a major differentiator between survivors and non-survivors, showing that lymphocyte counts were higher in survivors than non-survivors with (p = 0.105). Also, non-survivors had a higher median platelet count (278 × 10^3^/μL) compared to survivors (226 × 10^3^/μL); the difference did not reach statistical significance (p = 0.211). Albumin levels (p = 0.191) suggested that albumin may not be a major determinant of mortality. While the median CRP level was higher in non-survivors (p = 0.08), as shown in Table 2, indicating that CRP levels may not be a strong predictor of mortality. 

**Table 2:** Laboratory features of patients categorized by survivors and non-survivors. All the parameters are expressed in median-IQR. The statistical test used here is the Mann-Whitney test. IQR: interquartile range, HCT: hematocrit, PT: prothrombin time.

Laboratory characteristics	Reference range	Total (n = 100)	Survivors (n = 84)	Non-survivors (n = 16)	P-value
WBC (10^3^/uL)	4.0-11.0 × 10³/uL	9.51 (8.65-10.85)	9.32 (7.95-10.96)	12.31 (10.51-15.62)	0.001
Hemoglobin (g/L)	Males: 130-170 g/L, females: 120-150 g/L	130 (118-145)	128 (118-145)	121 (110-145)	0.046
Lymphocyte (10^3^/uL)	1.0-4.0 × 10³/uL	1.47 (1.11-1.91)	1.51 (1.13-1.92)	1.21 (1.02-1.47)	0.05
HCT (%)	Males: 40%-54%, females: 36%-48%	42.1 (38.2-45.1)	42.1 (38.2-45.1)	40.2 (37.9-46.2)	0.812
Platelet (10^3^/uL)	150-450 × 10³/uL	240 (206-290)	226 (205-305)	278(204-395)	0.211
Albumin (g/L)	35-50 g/L	41.6 (39.3-43.6)	41.8 (39.9-43.6)	40.7 (38.5-42.9)	0.191
CRP (mg/L)	<5 mg/L	4.1 (1.12-13.9)	3.2 (1.1-11.2)	8.2 (3.23-40.1)	0.08
PT (INR)	0.8-1.2 INR	1.09 (1.0-1.15)	1.09 (1.0-1.15)	1.11 (1.04-1.16)	0.361
Glucose random (mg/dL)	110–140 mg/dl	130 (104-178)	128 (103-177)	146 (115-192)	0.58

Using the HALP score demonstrated a notable difference between survivors (median 34.0) and non-survivors (median 24.0), with a p-value of 0.022. This suggests that a lower HALP score is associated with increased mortality in AIS. Length of stay (LOS) showed a significant difference, with survivors having a shorter median LOS (six days) compared to non-survivors (12 days), and the p-value was 0.012. ICU admission also revealed a highly significant difference between survivors (20 out of 84) and non-survivors (10 out of 16), with a p-value of <0.001, as shown in Table 3.

**Table 3:** HALP score of patients among survivors and non-survivors. HALP: hemoglobin, albumin, lymphocyte, and platelet, LOS: length of stay, ICU: intensive care unit. The statistical tests used here are the Mann-Whitney test and the Chi-square test.

Variables	Total (n = 100)	Survivors (n = 84)	Non survivors (n = 16)	P-value
HALP score (median)	31.2 (22.4–40.7)	34.0 (23.7–42.3)	24.0 (11.6–30.3)	0.022
LOS in days (median)	4 (4-8)	6 (5-10)	12 (6–15)	0.012
ICU admission (frequency)	30 (30)	20 (23.8)	10 (62.5)	<0.001

The findings of this study show that NIHS scores were categorized into mild, moderate, and high, representing the severity of stroke symptoms. Among the total population of 100 patients, those with mild NIHS scores (22) had 14 survivors and eight non-survivors. In the moderate NIHS score category (36), 31 were survivors and five were non-survivors. The high NIHS score category (42) included 39 survivors and three non-survivors. The distribution suggests a correlation between higher NIHS scores and increased mortality, as shown in Table 4.

**Table 4:** NIHSS scores of patients among survivors and non-survivors. NIHSS: National Institute of Health Stroke Scale. Mild: NIHSS score of 1-5, moderate: NIHSS score of 5-14, high: NIHSS score of 15-24.

NIHS (n)	Total (100)	Survivors (84)	Non-survivors (16)
Mild	22	14	8
Moderate	36	31	5
High	42	39	3

## Discussion

The HALP score serves as a comprehensive index reflecting components of the nutritional and immune status of patients. It has been previously demonstrated to play a prognostic role in various cancers, including gastrointestinal cancers, such as gastric cancer, esophageal squamous cell cancer, and advanced colorectal cancer [13]. However, in this research, we explored the correlation between the prognostic index HALP and patients diagnosed with acute ischemic stroke. A prognostic index is a tool utilized to predict the likely outcome or prognosis of a disease or condition. In this study, a total of 100 patients were included and divided into two categories according to mortality: 84 individuals survived, while 16 did not survive.

In this study, it was observed that patients in the non-survival group exhibited higher platelet counts (278 (204-395) × 10³/µL) compared to those in the survival group (226 (205-305) × 10³/µL), although this difference was not statistically significant (p = 0.211). Similarly, Yang et al. demonstrated that platelet count, particularly elevated platelets, could serve as a significant predictor for long-term outcomes in stroke patients, including recurrent strokes, mortality, and functional disability because platelets play a critical role in blood clotting and thrombosis, which are key factors in stroke pathophysiology and also elevated platelets are often associated with inflammation and vascular dysfunction [14]. Furthermore, additional findings of the present investigation pertained to hemoglobin levels. The hemoglobin level was slightly higher in the survivor group compared to the non-survivor group; however, this difference did not reach statistical significance (p = 0.46), indicating similar median values in both groups. Another study by Curtelin et al. [15] showed that low hemoglobin levels are associated with deteriorating neurological function in acute ischemic stroke (AIS) patients.

Another important component of the HALP score, albumin, particularly hypoalbuminemia, plays a significant role in predicting mortality in acute ischemic stroke. Although this study did not find a significant difference in albumin levels between the survivor and non-survivor groups, a systematic review by Thuemmler et al. delineates the importance of hypoalbuminemia in predicting poor post-stroke outcomes [16].

The severity of AIS, assessed by the NIHSS, indicated that among the 100 patients, 22 cases demonstrated mild severity (NIHSS score 1-5), 36 cases showed moderate severity (NIHSS score 5-14), and 42 cases presented with high severity (NIHSS score 15-24). This distribution suggests a correlation between higher NIHSS scores and increased mortality rates, suggesting that as stroke symptoms progress from mild to severe, survival prospects diminish. The results align with a previous study by Li et al. [17], indicating that a lower HALP score upon admission inversely correlated with the NIHSS score and was strongly linked to the occurrence of early neurological deterioration (END) within seven days. These findings echo earlier research suggesting a potential association between the systemic inflammatory cascade and malnutrition, which may worsen ischemic brain injury.

In this research, we observed a notable distinction in HALP scores between survivors (median 34.0) and non-survivors (median 24.0), with a statistically significant p-value of 0.02. This finding suggests that a lower HALP score is associated with increased mortality in this patient population. Our study's outcomes are consistent with previous research conducted by Peng et al. [18], who demonstrated a significant correlation between HALP score and survival in patients with bladder cancer. Additionally, Xu et al. [10] reported similar findings, indicating a relationship between HALP score, survival, and recurrence in patients with postoperative pancreatic cancer. Tian et al. [8] showed in their investigation that a higher HALP score is associated with a decreased incidence of recurrent stroke and mortality within 90 days and one year among individuals with AIS. Our results indicated a statistically significant contrast in NIHSS scores, HALP scores, and lymphocyte levels in relation to 28-day mortality among AIS patients. Soylu found that a low HALP score was a strong predictor of 28-day mortality in elderly AIS patients [4]. This study highlighted the practical utility of the HALP score in clinical settings to identify high-risk patients early.

The study has limitations; it was a single-center study, the sample size was small, and the measurement of the HALP score was taken only during the admission and was correlated with prognosis. The dynamic variation of the HALP score at various stages of the disease course was not studied. The follow-up period may not have been sufficient to capture all relevant outcomes, particularly long-term mortality and functional status beyond the study's timeframe. Longer follow-up could provide more robust data on the predictive value of the HALP score. Although the study adjusted for several confounding variables, there may still be unmeasured confounders that could influence the outcomes. Factors such as comorbid conditions, medications, and lifestyle factors were not fully accounted for. Multicenter research with a larger sample size will be required to confirm whether the HALP score is correlated with the prognosis of AIS. 

## Conclusions

This study underscores the potential of the HALP score as a prognostic biomarker in acute ischemic stroke (AIS) patients. Our findings reveal a significant association between lower HALP scores and increased mortality in AIS patients, indicating that a lower HALP score could serve as a valuable predictor of poor outcomes because clinicians could use it to identify patients at higher risk of poor outcomes, allowing for earlier intervention, more tailored treatment plans, and more informed discussions with patients and families about expected trajectories. Notably, survivors had higher median HALP scores compared to non-survivors, demonstrating its predictive capability. Additionally, the study highlights the importance of considering systemic inflammation and nutritional status in stroke prognosis. Despite the limitations, including the single-center design, small sample size, and limited follow-up duration, this research provides a foundation for future multicenter studies with larger cohorts to validate the HALP score's utility in clinical settings. Incorporating the HALP score into routine clinical practice may enhance the early identification of high-risk patients, potentially guiding treatment decisions and improving patient outcomes in AIS.

## References

[1] Murphy SJ, Werring DJ (2020). Stroke: causes and clinical features. Medicine (Abingdon).

[2] Shaikh F, Shaikh FH, Chandio SA (2021). Frequency of hypoalbuminemia and in-hospital mortality in acute ischemic stroke patients presenting at a tertiary care hospital, Hyderabad. Cureus.

[3] Patil S, Rossi R, Jabrah D, Doyle K (2022). Detection, diagnosis and treatment of acute ischemic stroke: current and future perspectives. Front Med Technol.

[4] Soylu VG (2023). Relationship between hemoglobin, albumin, lymphocyte, and platelet (HALP) score and 28-day mortality in very elderly geriatric critically ill patients with acute ischemic stroke. J Med Palliat Care.

[5] Esenwa CC, Elkind MS (2016). Inflammatory risk factors, biomarkers and associated therapy in ischaemic stroke. Nat Rev Neurol.

[6] Farag CM, Antar R, Akosman S, Ng M, Whalen MJ (2023). What is hemoglobin, albumin, lymphocyte, platelet (HALP) score? A comprehensive literature review of HALP's prognostic ability in different cancer types. Oncotarget.

[7] Zuo L, Dong Y, Liao X (2024). Low HALP (hemoglobin, albumin, lymphocyte, and platelet) score increases the risk of post-stroke cognitive impairment: a multicenter cohort study. Clin Intervent Aging.

[8] Tian M, Li Y, Wang X (2020). The hemoglobin, albumin, lymphocyte, and platelet (HALP) score is associated with poor outcome of acute ischemic stroke. Front Neurol.

[9] Konukoglu D, Uzun H (2017). Endothelial dysfunction and hypertension. Adv Exp Med Biol.

[10] Xu M, Chen L, Hu Y (2023). The HALP (hemoglobin, albumin, lymphocyte, and platelet) score is associated with early-onset post-stroke cognitive impairment. Neurol Sci.

[11] Xu SS, Li S, Xu HX (2020). Haemoglobin, albumin, lymphocyte and platelet predicts postoperative survival in pancreatic cancer. World J Gastroenterol.

[12] Papanagiotou P, Reith W, Kastrup A, Roth C (2014). Current reperfusion strategies for acute stroke. Interv Cardiol Clin.

[13] Shen XB, Zhang YX, Wang W, Pan YY (2019). The hemoglobin, albumin, lymphocyte, and platelet (HALP) score in patients with small cell lung cancer before first-line treatment with etoposide and progression-free survival. Med Sci Monit.

[14] Yang M, Pan Y, Li Z (2019). Platelet count predicts adverse clinical outcomes after ischemic stroke or TIA: subgroup analysis of CNSR II. Front Neurol.

[15] Curtelin D, Morales-Alamo D, Torres-Peralta R (2018). Cerebral blood flow, frontal lobe oxygenation and intra-arterial blood pressure during sprint exercise in normoxia and severe acute hypoxia in humans. J Cereb Blood Flow Metab.

[16] Thuemmler RJ, Pana TA, Carter B (2024). Serum albumin and post-stroke outcomes: analysis of UK regional registry data, systematic review, and meta-analysis. Nutrients.

[17] Li LL, Xie Y, Liang X (2023). Clinical application of HALP score to predict early neurological deterioration in elderly acute cerebral infarction patients. Research Square.

[18] Peng D, Zhang CJ, Tang Q (2018). Prognostic significance of the combination of preoperative hemoglobin and albumin levels and lymphocyte and platelet counts (HALP) in patients with renal cell carcinoma after nephrectomy. BMC Urol.

